# Data recovery methods for DNA storage based on fountain codes

**DOI:** 10.1016/j.csbj.2024.04.048

**Published:** 2024-04-24

**Authors:** Peter Michael Schwarz, Bernd Freisleben

**Affiliations:** Department of Mathematics and Computer Science, University of Marburg, Hans-Meerwein-Straße 6, Marburg, D-35043, Germany

**Keywords:** Fountain codes, Data recovery, Reconstruction, DNA storage, DNA, Data forensics

## Abstract

Today's digital data storage systems typically offer advanced data recovery solutions to address the problem of catastrophic data loss, such as software-based disk sector analysis or physical-level data retrieval methods for conventional hard disk drives. However, DNA-based data storage currently relies solely on the inherent error correction properties of the methods used to encode digital data into strands of DNA. Any error that cannot be corrected utilizing the redundancy added by DNA encoding methods results in permanent data loss. To provide data recovery for DNA storage systems, we present a method to automatically reconstruct corrupted or missing data stored in DNA using fountain codes. Our method exploits the relationships between packets encoded with fountain codes to identify and rectify corrupted or lost data. Furthermore, we present file type-specific and content-based data recovery methods for three file types, illustrating how a fusion of fountain encoding-specific redundancy and knowledge about the data can effectively recover information in a corrupted DNA storage system, both in an automatic and in a guided manual manner. To demonstrate our approach, we introduce DR4DNA, a software toolkit that contains all methods presented. We evaluate DR4DNA using both in-silico and in-vitro experiments.

## Graphical abstract

**Figure:**
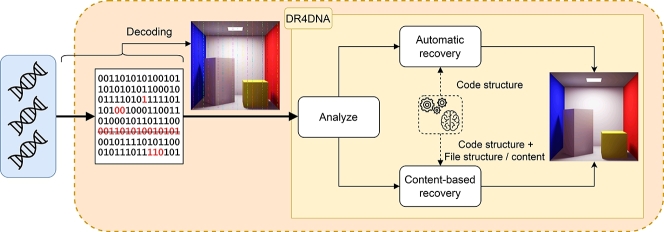

## 1 Introduction

The steadily increasing demand for large, environmentally sustainable, and long-term data storage solutions has directed considerable attention to DNA as a promising storage medium. To encode digital data in DNA, binary information is initially mapped to four DNA nucleotides: Adenine (A), Guanine (G), Cytosine (C), and Thymine (T). This mapping is usually performed using coding schemes that avoid error-prone DNA sequences, e.g., sequences with repetitive stretches of the same nucleotide (homopolymers) or an unfavourable proportion of G and C nucleotides (GC content). To enhance the resilience of data decoding in the presence of errors, redundancy is added in the form of an error-correcting code (ECC). Subsequently, the encoded data is synthesized using DNA synthesis methods, which typically yield small fragments (oligonucleotides; short: oligos) ranging from 40 to 100 base pairs (bp) [39]. The synthesized fragments are commonly stored *in-vitro*, but *in-vivo* storage is also an option [8]. The stored DNA fragments are read using DNA sequencing technologies. They generate text files that specify the order of the different nucleotides (nt) of the sequenced DNA strand and include information about the sequencing accuracy (i.e., base calling quality).

DNA synthesis, storage, and sequencing procedures are characterized by distinct error profiles and specific constraints [26], [39]. Typical constraints include a GC content between 40% and 60% both per sequence and in short intervals, as well as avoiding homopolymers longer than 3 or 4 nt. Additional constraints involve undesired motifs, which could include restriction sites used during the DNA synthesis process, biologically relevant motifs, or motifs that increase the probability of sequencing errors [27], [39].

The various restrictions that current and evolving DNA synthesis, storage, and sequencing methods impose on encoded digital data can be satisfied by fountain codes [10], [23], [29]. These codes treat synthesized DNA fragments as packets in a data stream, using an inner error-correcting code to protect each DNA fragment. If the inner code detects errors that it cannot correct, such as insertion and deletion errors (indels) or excessive substitutions, the individual DNA fragment is treated as an erasure. Erasures can be reconstructed from other DNA fragments using an outer fountain code. Fountain codes offer flexibility in generating numerous packets from input data, allowing users to tailor the outer code's rate based on anticipated error probabilities, intended applications, and resource constraints. Moreover, advanced inner codes can be deployed to detect and correct substitutions and indels up to a pre-defined threshold, and treat a packet as an erasure otherwise [48].

Fountain codes offer several benefits in the context of DNA storage [9], [10], [19], [48]. Due to the dependencies between the encoded packets, fountain codes support the propagation of repairs in the decoded data. The data encoded by fountain codes can be decoded in any order. Thus, it is not required to add indices to the data by the inner encoder. Instead, a *seed* is employed to sample a distribution function that determines the number of chunks (*n*) that will be XORed in each packet. The same seed is then used to select these *n* chunks. The decoder can reconstruct which chunks were used for each encoded packet by applying the seed to the same distribution function. Using this information, the decoder can reduce multiple packets to the original chunks using either belief propagation or Gaussian elimination with partial pivoting [25], [40]. The decoding process requires only (1+ϵ)⋅n correct symbols to decode the input data.

Basically, fountain codes are used to break the data to be encoded into chunks, from which many possible packets are generated. However, only a subset of these packets are required for decoding the data. Thus, fountain codes can replace DNA sequences that could not be synthesized or have a high error probability on-the-fly. They can be used to generate all possible DNA sequences, which can then be ranked based on their estimated error probabilities or entirely excluded based on factors such as GC content, homopolymer runs, or the likelihood of secondary structures, allowing the selection of the best n+ϵ elements.

Furthermore, fountain codes are less susceptible to the coupon collector's problem [40], minimizing the risk of decoding failures arising from missing portions of the encoded data after DNA sequencing.

However, since fountain codes are erasure codes, the underlying assumption is that each encoded packet is correct. Moreover, relying on belief propagation (or Gaussian elimination) for decoding lets even a single corrupted or missing packet disproportionately decrease the recoverable portion of the source data. DNA, as a biological storage medium, introduces the potential for errors, and a misinterpreted erroneous packet may transitively affect multiple sections of the decoded file.

Traditional digital data storage systems offer various solutions for data recovery in the event of medium failure. For hard disk drives, this typically involves the use of software tools for error prediction [49] and (file system) recovery [18], [28], as well as near-hardware procedures to retrieve otherwise inaccessible user data [44]. While recovering data from a failed hard disk drive is generally possible, any error exceeding the chosen maximum redundancy for a DNA storage system leads to irreversible data loss. Considering the significance and enduring nature of data stored in DNA, the absence of data recovery mechanisms is a major drawback of DNA as a storage medium.

In this article, we present a novel approach to provide data recovery for DNA storage. Compared to the common use of forward error codes to automatically correct errors introduced by the DNA storage process, we provide an additional layer of recovery, comparable to recovery of data on partially destroyed hard disk drives. In particular, we present a novel method for automatically recovering corrupted data stored and encoded in DNA using fountain codes. Our method is not limited to any specific file type and exploits the fountain code structure of the encoded data to detect and correct corrupted data. Moreover, we present file type-specific and content-based data recovery methods for three file types. Utilizing file type-specific information and general knowledge about the stored data is not exclusive to fountain codes, but the knowledge introduced by the fountain encoding can be effectively combined with file type-specific redundancy and general knowledge about the stored data to successfully reconstruct information from a partially corrupted DNA storage system. Our file type-specific and content-based recovery methods represent a fallback recovery approach in cases where our encoder-based method does not recover a file completely – this is comparable to data recovery services for hard disk drives.

In addition, we facilitate the utilization of external data recovery tools in conjunction with the proposed methods. Furthermore, we illustrate how the proposed methods can be employed to retrieve the stored information when not all data is present. This bridges the gap towards conventional data forensics for the DNA storage use case. We demonstrate the feasibility of our approach by DR4DNA, a software toolkit that includes all the presented methods for the reconstruction of missing or corrupted data encoded in DNA with fountain codes. We evaluate the proposed methods implemented in DR4DNA using both in-silico and in-vitro experiments.

Since in our previous work on NOREC4DNA [40] Raptor codes [43] outperformed other fountain codes, we use Raptor codes in all of our experiments. However, the presented methods can be applied to any en-/decoding scheme using fountain codes, including the approach of Erlich and Zielinski [10] based on the Luby transform code [23] and Online codes [29], both of which we implemented in NOREC4DNA. Furthermore, although generally requiring a higher computation time than belief propagation, we use Gaussian elimination to formalize the decoding of data encoded using any fountain code. Therefore, the time complexity for decoding each input is $O(nm^{2})$, where *n* is the number of rows and *m* is the number of columns. The space complexity of Gaussian elimination is O(nm), where *m* is the number and *n* is the size (in bytes) of the input packets. When performing automatic repair, the time and space complexity are influenced by the number of permutations (*i*) computed: the time complexity is $O(inm^{2})$ and the space complexity is O(inm).

All algorithms described below can also be implemented using a belief propagation or inactivation decoder approach. When using belief propagation for decoding as described by Shokrollahi [43] and Etesami and Shokrollahi [11], the time complexity is linear in the number of packets parsed (i.e., O(nlog⁡(1/ϵ)), where *ϵ* is the overhead). The content-based methods add complexity by additionally parsing the decoded data. However, this typically requires a single linear scan over the decoded data. Note that all of our proposed methods are only executed if the initially used fountain code fails to successfully decode the stored file(s).

In summary, our contributions are as follows:

• We present a novel decoder-based data recovery method to reconstruct corrupted or missing data encoded in DNA using fountain codes. It takes advantage of the fountain code structure of the encoded data and can be combined with our proposed file type-specific and content-based data recovery methods.
• We distinguish between three operational recovery modes: (1) file-independent automatic recovery by using only the inconsistencies in the fountain encoding, (2) automatic recovery using file type-specific information and general knowledge about the stored data, and (3) guided manual recovery using both file type-specific information and the general knowledge about the stored data.
• We present an implementation of all of these methods in our novel software toolkit DR4DNA to demonstrate their advantages and real-world usability. We make the code of DR4DNA available to the public with an open source license.
• We evaluate the proposed methods implemented in DR4DNA using both in-silico and in-vitro experiments.
• We discuss how to use the presented methods to improve DNA storage systems based on fountain codes.

## 2 Material and methods

### 2.1 Overview

We present a method for automatically detecting and correcting linearly independent errors. Traditional error detection methods, such as packet-based or file-wide error detection, use additional symbols either added to each encoded packet or as additional information (e.g., in the form of a checksum in a header). Such error detection and correction methods are commonly used in several approaches related to DNA data storage, such as Hedges by Press et al. [35], forward error correction for DNA data storage by Blawat et al. [3], the coding scheme used for robust chemical preservation in DNA by Grass et al. [14], DNA fountain by Erlich and Zielinski [10], NOREC4DNA by Schwarz and Freisleben [40], hidden addressing encoding for DNA storage by Wang et al. [46], and DNA-Aeon by Welzel et al. [48]. While the first three approaches use conventional coding schemes with error detection and correction methods, the last four approaches incorporate fountain codes to encode digital information for the DNA storage channel. Our approach is based on dependencies between decoded chunks using any fountain code decoder. Therefore, our proposed methods are limited to encoding schemes using fountain codes. Since these methods solely rely on the fountain coding structure as well as (optionally) on the structure and content of the stored files, our methods can be used without changes to the fountain coding structure, allowing error recovery comparable to existing solutions for conventional data storage systems, such as hard drives. We explain the specific conditions required to achieve a fully automated repair below. Furthermore, we outline various approaches for performing file type-specific and content-based analysis to correct errors in situations where the aforementioned conditions are not satisfied.

The presented methods are primarily intended to be implemented, integrated, and extended by computer scientists or bioinformatics researchers in the realm of coding schemes for DNA data storage, but the tools provided in the DR4DNA software toolkit are designed to be used by any researcher interested in DNA data storage.

### 2.2 Decoder-based automatic error detection and correction

Fountain codes operate on unordered packets with near-optimal properties to support the mitigation of packet erasures. Apart from adding additional copies of the encoded data through repeated synthesis [13] or PCR for physical redundancy, it is common practice for DNA data storage to create and store more packets than required to add logical redundancy [10], [40]. Our method uses these additional encoded packets as the basis for automatic repair. The proposed method is limited to errors in packets that would leave the linear equation system of the decoder in an inconsistent state. It can detect an error as well as its delta to the original data to correct the error, even in the absence of additional dedicated error correction symbols. Thus, the proposed method introduces no additional overhead compared to what is required for the successful erasure channel-aware error-free decoding. It exploits the inherent properties of the fountain code structure. The basic mathematical principles of our method are presented below.

Theorem 1 *The encoded packets of a fountain code can be defined as an (over-determined) linear equation system (* M×N *). Assuming the absence of errors and the linear independence of N packets, this linear equation system has exactly a single solution.*

Proof Each encoded packet is a non-trivial (degree ≥1) linear combination of a subset of the input and intermediate symbols in GF(2). The intermediate symbols can be defined as a linear combination of the input symbols in GF(2). The input symbols *s* can be defined as $I_{N}x=s$. To reconstruct the source symbols, belief propagation or Gaussian elimination can be used on the set of encoded symbols $\left(A_{p},b_{p}\right)$.

Given that the intermediate symbols are algebraically derived (non-trivially) from the source symbols, and the encoded symbols are defined as a set of non-trivial linear combinations, the rank *r* of the generated encoded packets cannot surpass $N=rank(I_{N})$, i.e., the rank of the input symbols.

Utilizing the Rouché–Capelli theorem [42], there are two cases for an equation system $Ax=b\text{ with }A\in F_{2}^{M\times N},k=rank(A)\text{ and }l=rank([A|b])$:

• Case 1: k<l: The equation system is inconsistent and thus does not have a valid solution.
• Case 2: k=l: There is at least one solution. If k=n, the solution is unique and the equation system is *solved*. However, if l=k<n, then there are n−k free parameters, resulting in infinitely many solutions.

Since by definition the rank *l* of the augmented matrix [A|b] must be greater or equal than rank(A), there is no case with k>l. Therefore, fountain codes with *N* linearly independent packets have exactly a single solution. □

Lemma 2 *An error in a packet* $p_{i}$ *may increase the rank* $r^{\prime }=rank([A^{\prime }|b^{\prime }])$ *of a linear equation system derived from packets received by a fountain code, compared to the error-free version with* r=rank([A|b]) *. Assuming* $p_{i}$ *corresponds to row* $\left(A_{i}^{\prime },b_{i}^{\prime }\right)$ *, then* $r^{\prime }=r+1$ *if* $\left(A_{i},b_{i}\right)$ *can be algebraically derived from the remaining rows, but* $\left(A_{i}^{\prime },b_{i}^{\prime }\right)$ *cannot be derived from the remaining rows.*

Since fountain codes operate on packets of equal length, we can directly detect insertions and deletions and remove such packets from the input of the decoder. A series of insertions and deletions resulting in a sequence of correct length can thus be described as having *n* substitutions, where *n* is the Hamming distance between the original and the modified sequence. If a substitution changes a packet $p_{i}=(a_{i},b_{i})$, it might modify either $a_{i}$, $b_{i}$, or both. Using a seed to infer the used packets, $a_{i}$ will be modified if the substitution alters the seed.

Lemma 3 *Since for all possible values of* $a_{i}$ *a valid version of* $b_{i}$ *exists, a corrupted packet* $p_{i}=(a_{i},b_{i})$ *encoded using a fountain code can be treated as having the error only in the* $b_{i}$ *portion.*

While the Rouché–Capelli theorem shows that an inconsistent equation system does not have a valid solution, we can still apply algorithms for solving linear equations, such as Gaussian elimination, to generate potentially incorrect results. In this context, the result $b_{r}$ is defined as the first *N* rows of *b* after the Gaussian elimination transformed [A|b] into its reduced row echelon form.

Theorem 4 *Solving an inconsistent equation system* Ax=b *with* $A\in F_{2}^{M\times N}$ *,* rank(A)=N *, and* rank(A)<rank([A|b]) *using Gaussian elimination (or belief propagation) will yield different results based on the order of the rows and the algorithm used.*

Proof Assuming that the equation system Ax=b is inconsistent, then there exists a subset *S* of rows $\left(A_{s},b_{s}\right)$ with $rank(A_{s})<rank([A_{s}|b_{s}])$ with: ∃i,red where $red(A_{s}\setminus A_{s_{i}})=A_{s_{i}}$ but $red([A_{s}|b_{s}]\setminus [A_{s_{i}}|b_{s_{i}}])\neq [A_{s_{i}}|b_{s_{i}}]$. Here, *i* is an index of *A* in *S* and x↦red(x) is a series of multiplications of rows in *x* applied to a (neutral) null row ($\overset{\to }{0}$). Therefore, two contradicting solutions for $b_{s_{i}}$ exist: either using a reduction path including $A_{s_{i}}$, or using the series of row multiplications of *red* as described above. Depending on the order of the elementary row operations to solve Ax=b, there are three options for a non-trivial function *red* to reduce each row of *A* to its version in the reduced row echelon form of *A* (and the corresponding line in *b*) for the equation system:

(1) *red* does not use any elementary row operations using a row from any unique subset fulfilling the definition of *S* above: the resulting row was derived from rows that are not part of any existing contradiction in *A* and thus the order of elementary row operations does not influence the row.

(2) *red* contains an elementary row operation using $\left[A_{s_{i}}|b_{s_{i}}\right]$ of a subset *S*: the resulting row $b_{r}$ will be influenced by $b_{s_{i}}$.

(3) *red* uses a series of elementary row operations from $\left[A_{s}|b_{s}]\setminus [A_{s_{i}}|b_{s_{i}}\right]$: the resulting row $b_{r}$ will be reduced using the contradicting version of $b_{s_{i}}$.

While option (1) is only possible if no row in any set of rows with inconsistencies was used in *red*, options (2) and (3) are possible for each row reduced using any row in any set of rows with inconsistencies. Therefore, for an inconsistent equation system with rank(A)<rank([A|b]) as defined above, the reduction path influences the resulting system. □

In Example 1, we illustrate Theorem 4. The example demonstrates that altering the sequence of rows in an inconsistent equation system yields a differing outcome.

Example 1 (1) $$Ax=b⟷\left(\begin{array}{ccc} 1 & 0 & 0\\ 0 & 1 & 1\\ 0 & 0 & 1\\ 0 & 1 & 0 \end{array}\right)x=\left(\begin{array}{c} 110001\\ 011110\\ 001011\\ 010110 \end{array}\right)$$

After Gaussian elimination (row 2 = row 2 + row 3):

(2) $$A^{\prime }x=b^{\prime }=\left(\begin{array}{c} 110001\\ 010101\\ 001011\\ 010110 \end{array}\right)$$

where $A^{\prime }$ is the matrix *A* after the algorithm finished. It has the form $A^{\prime }={\left(I_{3},a_{4}^{\prime }\right)}^{⊺}$ However, if we augment the order of rows of *A* by swapping row 2 and 4 to create $A_{aug}$, we get:

(3) $$A_{aug}x=b⟷\left(\begin{array}{ccc} 1 & 0 & 0\\ 0 & 1 & 0\\ 0 & 0 & 1\\ 0 & 1 & 1 \end{array}\right)x=\left(\begin{array}{c} 110001\\ 010110\\ 001011\\ 011110 \end{array}\right)$$

After Gaussian elimination (trivial, since no elementary row operation has to be performed):

(4) $$A_{aug}^{\prime }x=b_{aug}^{\prime }=\left(\begin{array}{c} 110001\\ 010110\\ 001011\\ 011110 \end{array}\right)$$

Since $b^{\prime }\neq b_{aug}^{\prime }$ (as indicated by Equations (2) and (4)), we get differing results for the second variable. It is evident that the linearly independent rows, such as row 1 of *A*, can be swapped with any other row without changing the result.

By adding an identity matrix to the input [A|b] of the Gaussian elimination to create $\left[A|I^{M\times M}|b\right]$, the resulting augmented matrix not only yields the solved input data $b^{\prime }$, but also $A^{-1}$ (more precisely, a left inverse of *A*; a general inverse could be retrieved by creating a n×n-matrix of the received data such that it has a rank of *n*). Each column *j* of a row *i* in this inverse can be interpreted as the contribution of the corresponding row *j* in *A* to the creation of the output row *i*.

Definition 1 A packet is called non-critical when removing its row from the equation system Ax=b does not result in a reduction of the system's rank r=rank([A]). Otherwise, it is called critical. For a (single) corrupted packet, this is equivalent to rank(A)<rank([A|b]) (as described in Theorem 4) when the packet is non-critical.

Theorem 5 *For a solved but erroneous equation system derived from a set of packets encoded using fountain codes, the index i of a corrupted packet in A can be determined by annotating a sufficient number of rows in* $b^{\prime }$ *as correct or incorrect using:* $S_{\mathrm{corrupt}}=\cap_{r\in b_{e}}r\setminus \cup_{r\in b_{c}}r=\{i\}$ *.*

Proof Using $A^{-1}\in F_{2}^{M\times N}$ as defined earlier, and the sets $b_{c}$ and $b_{e}$ (as the sets of indices of correct and erroneous rows in $b^{\prime }$) as well as assuming that there exists only one corrupted packet with index *i*, we define to simplify: $S_{r}=\{j\in A^{-1}:A_{j}^{-1}r=1\}$. For each row r∈{0..M}, $S_{r}$ is a set of indices indicating which rows of *A* (and *b*) were used during elementary row operations to produce $A_{r}^{\prime }$ (and $b_{r}^{\prime }$). With $b_{c}$, $b_{e}$ and $S_{i\in 0\ldots N}$ as the vector of sets including all used packets for each row *i*, we can calculate the set of indices of possibly incorrect packets as follows.

For the subset of erroneous rows: $\exists b_{e}:i\in \cap_{r\in b_{e}}r$. Utilizing $b_{e}=\{j\in \{0\ldots M\}:A_{ji}^{-1}=1\}$, this is trivial. Furthermore, using $b_{c}$: $∄b_{c}:i\in \cup_{r\in b_{c}}r$. By contradiction, if *i* were in $b_{c}$, there would exist a correct row *r* in $b^{\prime }$ with $i\in S_{r}$ (reduced using the corrupted row *i*). ↯

Considering that each row (the first *N* if *A* is over-determined) of a solvable linear equation system must have a unique row in $A^{-1}$ (otherwise, the matrix would not have a full rank), we deduce: $\forall i\in \{0\ldots M\},\exists b_{c},b_{e}:\{i\}=\cap_{r\in b_{e}}r\setminus \cup_{r\in b_{c}}r$. □

Lemma 6 *If there are multiple corrupted packets (denoted as a set* $S_{\mathrm{corrupt}}$ *using their indices in* $A^{\prime }$ *), the formula of* Theorem (5) *requires modification:* $S_{\mathrm{corrupt}}\subseteq \cup_{r\in b_{e}}r\setminus \cup_{r\in b_{c}}r$ *.*

Although the calculation of corrupted packet(s) remains feasible, utilizing the set union for $b_{e}$ instead of the intersection will generally require tagging more packets to reduce the resulting set of corrupted packets to the correct number of packets.

Knowing the corrupted packet $p_{i}$, a repair of the decoded file can be performed by correcting a single incorrect row *j* in $b^{\prime }$ that was affected by $p_{i}$ during decoding ($A_{j,i}^{-1}=1$). Using $b_{j_{\mathrm{corrected}}}^{\prime }$ as the row $b_{j}^{\prime }$ after correction, a formal approach to propagate this change involves computing $b_{\mathrm{corrected}}=A^{-1}b_{\mathrm{corrected}}^{\prime }$ and subsequently solving $Ax=b_{\mathrm{corrected}}$. Alternatively, using the difference (in GF(2)) denoted as $\delta_{i}=b_{j_{\mathrm{corrected}}}^{\prime }⊻b_{j}^{\prime }$, the corrected version for all chunks *k* reduced using the corrupted input can be calculated as follows: $b_{k_{\mathrm{corrected}}}^{\prime }=b_{k}^{\prime }⊻\delta_{i}$ with $k\in S_{i}$. Additionally, it is possible to use the difference to directly repair the initially corrupted packet by calculating $b_{i}=b_{i}⊻\delta_{i}$ on the initial *b* and then performing a decoding run using Gaussian elimination or belief propagation.

Given that all chunks in $\left\{b_{j}^{\prime }\text{ with }i\in S_{j}\right\}$ can be used to calculate $\delta_{i}$, a user can select the decoded chunk that is the easiest to repair and subsequently propagate $\delta_{i}$ to all other chunks affected by the corrupted packet $p_{i}$.

Given a single corrupted linearly dependent packet, the impact of altering the order of rows in the equation system on $b^{\prime }$ can be described through Theorem 4. Stated formally:

Theorem 7 *With all chunks* $b_{i}^{\prime }$ *being reduced using the corrupted packet* $p_{j}$ *having the same error* $\delta_{j}$ *compared to their initially encoded version* $\delta_{j}=b_{i}^{\prime }⊻b_{i_{org}}^{\prime }$ *, the original file can be reconstructed if a non-zero difference between multiple versions of the same (decoded) chunk can be computed.*

Proof Let $\mathrm{per}m_{\mathrm{all}}$ be the set of all possible permutations of the equation system [A|b], and $\mathrm{per}m_{\mathrm{diff}}$ be a subset containing permutations of [A|b] that yield a different result $b^{\prime }$ for the first *n* rows of $b^{\prime }$, where *n* is the number of chunks the original file has been split into.

For each version *i* of a row $b_{i}^{\prime }[k],0\leq k\leq n$ derived from a permuted version of the equation system compared to all other versions *j* (where j≠i) of $b_{j}^{\prime }[k]$ for this row, the difference can be calculated as follows: $\delta_{ij}[k]=b_{i}^{\prime }[k]⊻b_{j}^{\prime }[k]$. Additionally, the difference of the used packets during the reduction of these chunks can be computed as: ${p\_\mathrm{diff}}_{ij}[k]=A_{i}^{-1}[k]⊖A_{j}^{-1}[k]$, as well as the common packets: ${p\_\mathrm{eq}}_{ij}[k]=A_{i}^{-1}[k]\cap A_{j}^{-1}[k]$. Starting with β={0…n} and α={}, we can calculate *β* as the set of corrupted packet candidates and *α* as the set of known correct packets:

(5) $$\alpha =\cup_{i\in \mathrm{per}m_{\mathrm{diff}}}\cup_{j\in \mathrm{per}m_{\mathrm{diff}}\setminus i}\cup_{k\in [1\ldots n],\delta_{ij}[k]\neq 0}{p\_\mathrm{eq}}_{ij}[k]\cup \cup_{i\in \mathrm{per}m_{\mathrm{diff}}}\cup_{j\in \mathrm{per}m_{\mathrm{diff}}\setminus i}\cup_{k\in [1\ldots n],\delta_{ij}[k]=0}{p\_\mathrm{diff}}_{0j}[k]$$

Note that in Line 1 of Equation (5) we use packets that were used in both permutations *i* and *j* to create differing solutions ($\delta_{ij}[k]\neq 0$). If there is only one corrupted packet, as the solutions differ, all packets used to reduce both solutions must be correct.

The reduced set of possible corrupted packets can be calculated as follows:

$$\beta =\cap_{i\in \mathrm{per}m_{\mathrm{diff}}}\cap_{j\in \mathrm{per}m_{\mathrm{diff}}\setminus i}\cap_{k\in [1\ldots n],\delta_{ij}[k]\neq 0}{p\_\mathrm{diff}}_{ij}[k]\setminus \alpha$$

Since, by definition, there exists a non-zero difference and thus at least two differing versions of $b^{\prime }$, Theorem 5 can be applied. All chunks derived from the packets in *β* are considered invalid, and all others (derived from packets in α∖β) are deemed valid. Using the knowledge about the corrupted packet as well as the difference $\delta_{j}$, the packet can be corrected and thus the originally encoded information can be retrieved. □

Given the existence of differing solutions, using Theorem 5, Theorem 7, we can calculate the corrupted packet and its difference to the correct solution as shown in Algorithm 1.

**Algorithm 1:**
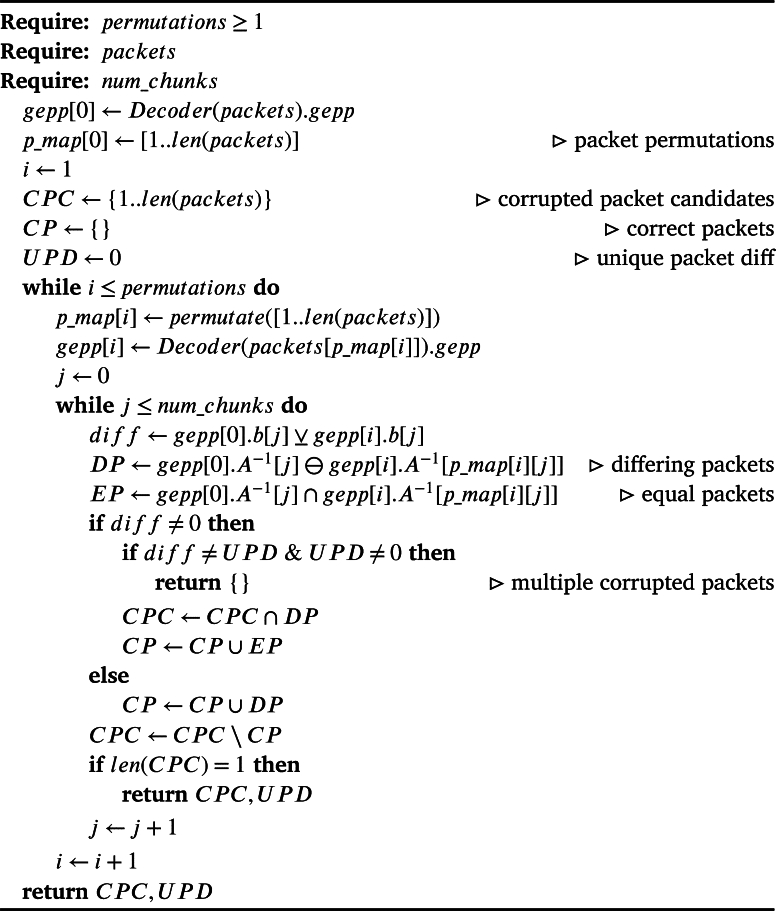
Finding a corrupted packet using permutations of the input

If there is more than one corrupted packet, all corrupted packets that adhere to these restrictions can be detected and repaired:

• the corrupted packets have a linear dependency with correct packets (and thus create differing solutions)
• the errors (differences $\delta_{i}$ to the correct rows) must not be linearly dependent

Additionally, it is no longer safe to assume that all errors propagated to the erroneous result of the initial decoding; thus, either a user would have to decide which differing version of a chunk (and thus a packet) is the correct one, or a global checksum (as mentioned earlier) has to be used. Since, as already mentioned in the proof of Theorem 7 regarding Equation (5) Line 1, this step of the calculation can only be performed if there is only one corrupted packet (one difference). Therefore, we would either have to omit this step or perform a series of prior steps as shown below. For this purpose, we calculate the set *γ* of all (unique) differences:

$$\gamma =\cup_{i\in \mathrm{per}m_{\mathrm{diff}}}\cup_{j\in \mathrm{per}m_{\mathrm{diff}}\setminus i}\cup_{k\in [1\ldots n]}\delta_{ij}[k]$$

This set might include linearly dependent differences that are the result of a row being reduced using multiple corrupted packets. To filter out all linearly dependent differences, two assumptions are used: (1) linearly dependent differences (derived from the same two or more corrupted packets) occur less often than errors introduced by only a single packet, and (2) the number of differing bytes is higher for errors introduced using multiple corrupted packets compared to such with only one corrupted packet. By sorting all collected differences *γ* according to these two metrics, it is possible to detect and filter any difference (*δ*) introduced using a combination of multiple corrupted packets with a high probability. This will yield $\gamma^{\prime }$ as the subset of unique and linear independent differences. To calculate the corrupted packet for each unique and linear independent difference in $\gamma^{\prime }$, the steps described above (Theorem 7 and in Algorithm 1 respectively) have to be performed for each unique error $\delta_{l}\in \gamma^{\prime }$:

(6) $$\alpha_{l}=\cup_{i\in \mathrm{per}m_{\mathrm{diff}}}\cup_{j\in \mathrm{per}m_{\mathrm{diff}}\setminus i}\cup_{k\in [1\ldots n],\delta_{ij}[k]=l}{p\_\mathrm{eq}}_{ij}[k]\cup \cup_{i\in \mathrm{per}m_{\mathrm{diff}}}\cup_{j\in \mathrm{per}m_{\mathrm{diff}}\setminus i}\cup_{k\in [1\ldots n],\delta_{ij}[k]=0}{p\_\mathrm{diff}}_{0j}[k]\beta_{l}=\cap_{i\in \mathrm{per}m_{\mathrm{diff}}}\cap_{j\in \mathrm{per}m_{\mathrm{diff}}\setminus i}\cap_{k\in [1\ldots n],\delta_{ij}[k]=l}{p\_\mathrm{diff}}_{ij}[k]\setminus \alpha_{l}$$

Additionally, we could utilize all previously detected linearly dependent differences. For this purpose, we need to modify the restriction $\delta_{ij}[k]=l$ to: $\exists E\subseteq \gamma^{\prime }:\delta_{ij}[k]={⊻}_{e\in E}e\text{ and }l\in E$ (considering all pairs of rows, which produce a difference that can be represented as a linear combination of errors, where one of the errors is the current target difference *l*.) This yields a set of pairs $\left\{\delta_{l},\beta_{l}\right\}$, where $\delta_{l}$ is the error introduced through the packet $\beta_{l}$. As long as all corrupted packets have a different linear dependency (and thus produce a non-trivial $\delta_{l}$), the packets can then be independently repaired, and thus the originally stored content can be reconstructed. An example for the automatic correction of multiple errors can be found in the supplement.

### 2.3 File type-specific and content-based error detection and correction

While the decoder-based method presented in the previous section can automatically detect and repair corrupted chunks under certain conditions, these conditions might not always be fulfilled. Since a larger overhead of encoded packets increases the chance of having multiple valid convergence paths, as discussed in Section 3.3, the probability of success for the decoder-based method decreases with decreasing overhead. To further increase the chance of a successful repair, it is possible to combine file type-specific and content-based information with the knowledge gained from the encoding to detect errors and thus possibly corrupted packet(s). Although this approach requires knowledge about the stored information and might thus not be sufficient to perform a fully automatic recovery, it can assist a user in finding a corrupted packet as well as the error delta with which a repair can be performed. By reducing the amount of possibly corrupted packets and error deltas in such a way, generating all remaining possible combinations for the encoded file can be a viable solution. If combined with a file-wide checksum, this can then even be used to automatically retrieve the originally encoded file.

To leverage file type-specific and content-based information, we use an error matrix to indicate if a specific byte in the decoded file is valid, of unknown status, or invalid. In the case of an invalid byte, the file type-specific and content-based analysis may additionally provide information about the correct data and thus the error, represented as the delta between the correct and actual byte. This approach operates at a byte level, offering a more granular perspective compared to the manual tagging of entire chunks as valid or invalid. However, this granularity may result in a reduction of information gained for each chunk, since a chunk with a mixture of valid and unknown status bytes will be classified entirely as having an unknown state. Since the file type-specific and content-based reconstruction can use the cross-chunk dependencies introduced by the packets used during decoding, even a partially filled byte-level error matrix can substantially reduce the set of possibly corrupted packets or even enable a fully automatic recovery.

To demonstrate the flexibility of our file type-specific and content-based approach, we implemented it as a proof-of-concept for different file types.

#### 2.3.1 Plain text files

To analyze plain text encoded using fountain codes, an initial step involves determining the language of the stored data using a natural language processing algorithm. This can be performed either by a statistical approach like ‘Language-Detection’ [6], the N-gram based text categorization integrated into ‘NLTK’ [5] or with the help of libraries using translation services such as Google Translate, known for accurate language detection [17]. Once the correct language is identified, the grammar and spelling checker LanguageTool [33] can be used to construct an array of the most probable correct characters for the decoded string. While this may successfully repair the complete message, it is essential to note that it may only partially recover the encoded information or potentially introduce new errors, particularly if the encoded string deviates from existing grammar and language rules. Combining the results of the grammar and spelling checker with the information introduced by the equation system can help to mitigate these challenges. Due to the fixed length of each chunk in the decoded solution, the length of the resulting string and more precisely the length of each chunk is guaranteed to remain constant. This restriction limits the search for a corrected equation system by only including solutions that yield a row with the same length and have the minimal possible Levenshtein distance [22] compared to the decoded sequence.

**Fig. 1:**
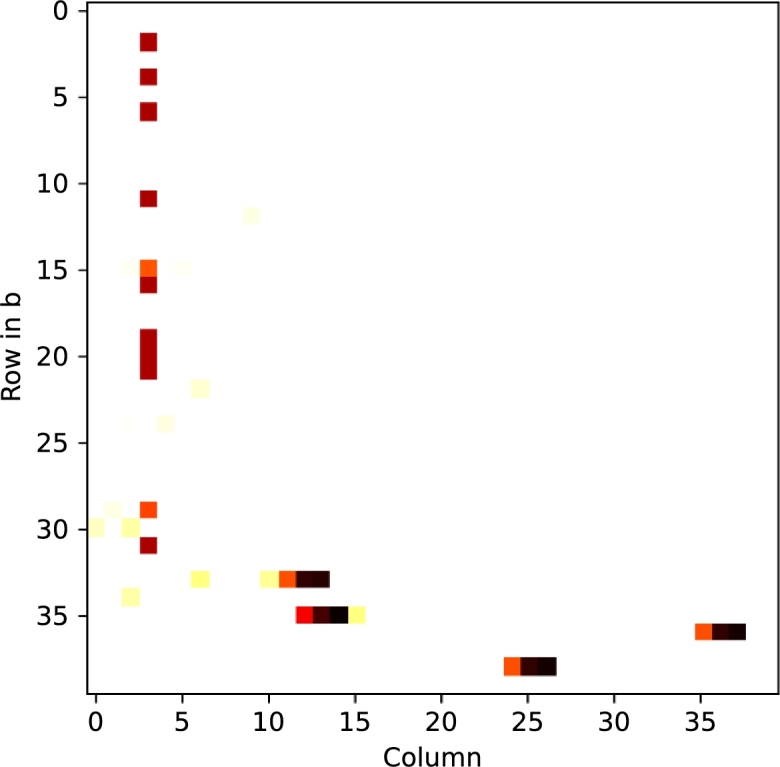
Heatmap of the first 40 rows of the calculated error matrix. The matrix is derived from the error delta between the actual result (*b*′) and the version repaired using our LanguageTool-based maximum likelihood correction approach. Each column's color represents the detected error delta. In this representation, all (non-white) positions with the same color in the same column might indicate an error, while white fields in the same column indicate correct rows. Although there are various locations with a non-zero error delta, column 3 exhibits a notably higher number of errors, most of which have the same error delta. Therefore, the error position and the most common error delta can be clearly distinguished from non-aligning false-positives.

To address false-positive corrections, position-wise relations introduced during encoding are incorporated by creating a difference-matrix $b_{\delta }^{\prime }=b^{\prime }⊻b_{c}^{\prime }$ between the decoded data $b^{\prime }$ and the corrected version $b_{c}^{\prime }$, as shown in Fig. 1.

**Fig. 2:**
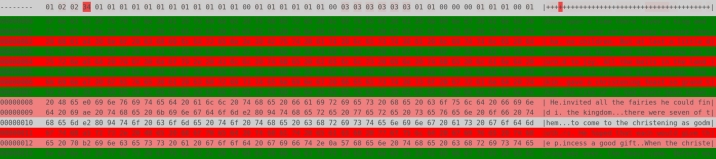
Automatic error detection using the LanguageTool. Rows exceeding a predefined threshold are automatically categorized as correct or incorrect. The first row shows the number of errors found for each column. Although the LanguageTool detected errors in almost all columns, the column at which the actual error was introduced exhibits 34 identical error deltas, as detected by the plugin.

Sorting the differing (non-zero) values for each column by their number of occurrences in this matrix yields the most common error deltas for each position. Since a corrupted packet introduces errors at the same position in each affected row, this eliminates invalid corrections introduced by the spelling check and leaves only such systematic errors. Using these per-column error deltas (or for simplicity, the most common per-column error $\delta_{j}$), we can infer the corrupted packet as described in Theorem 5. Treating every row *i* with $b_{\delta_{ij}}^{\prime }=\delta_{j}$ as corrupted and either all others as correct or only those with $\delta_{j}=0$ (indicating no error) helps to reduce the number of possibly corrupted packets. If multiple columns have a non-zero $\delta_{j}$ above a selected threshold, this process can either combine multiple columns to identify a corrupted packet with multiple corrupted bytes or perform this procedure for all such columns *j* exceeding the threshold. In the latter case, the most likely correction and $b_{\delta }^{\prime }$ are recalculated in each iteration. To illustrate this approach, we encoded a text file containing the *Sleeping Beauty* fairy tale into 40-byte chunks and introduced a substitution error into an encoded sequence. The result of the automatic error tagging is depicted in Fig. 2.

We presented a systematic recovery process to (1) identify corrupted columns, (2) optionally configure the target number of columns for repair, (3) automatically tag correct and incorrect rows, and (4) automatically repair decoded data by propagating the difference $\delta_{j}$ to all packets affected by the corrupted packet. If step (3) leaves multiple possibly corrupted packets, a user might have to tag additional rows as correct or incorrect, or choose to create all possible results.

For raw text sequences that conform to most grammar and spelling rules, this correction method reliably corrects errors. Using more sophisticated text analysis methods might even allow us to recover the encoded data if there are missing packets that prevent a full decoding. For instance, employing token-based language models like ChatGPT [45] could infer missing chunks and use them to decode rows still constrained by multiple packets. However, this approach is beyond the scope of this article.

#### 2.3.2 BMP images

In addition to text files, our framework supports the BMP bitmap image format. Currently, our implementation lacks support for advanced features like JPEG/PNG compression methods, but we plan to include such features in the future. Intially, the decoded data is parsed using a customized Kaitai Struct parser [36] for the BMP file format. This parser is adapted to add a start and end position to each parsed value, as well as to automatically detect and repair invalid static or conflicting information. For BMP files, this capability is limited to the various headers and their corresponding fields.

The ‘DIB-header’, the actual ‘pixel array’, and the ‘bitmap file header’ (shown in Table 1) are the only three non-optional headers that we will focus on below.

**Table 1:** Structure of the bitmap file header

Offset	Size (bytes)	Description
00	2	Signature (BM, BA, CI, CP, IC, PT)
02	4	Size of the BMP file
06	2	Reserved
08	2	Reserved
0A	4	Starting address of the pixel array

While the default behavior of the employed parser would encounter a failure upon encountering an illegal symbol, we modify the process to create a reconstructed version $b_{r}^{\prime }$, where any byte that would produce a failure during parsing will be replaced by the expected value. If there are multiple possible values for such a byte (e.g., the header field), the replacement with the smallest Levenshtein distance is selected. Alternatively, users have the option to manually modify these values. Leveraging the known length of the data decoded using fountain codes, it is possible to infer various other variables for a BMP file. Parsing all header regions, including width, height, and the number of bytes per pixel, allows us to correlate these values with the start position of the pixel array. Depending on the BMP implementation, the ‘DIB-header’ may contain a different number of fields including size of the header, width, height, number of planes (must be 1), and number of bits per pixel (e.g., for the ‘OS/2 1.x bitmapcoreheader’) [7].

Due to the design choice of padding each row in the pixel array to a multiple of 4 bytes in size, and the fact that the length of such a row does not necessarily have to align with the length of a decoded chunk, additional known data for each row in the bitmap is potentially obtained. Each of these rows may start at a different position in the decoded matrix $b^{\prime }$.

**Fig. 3:**
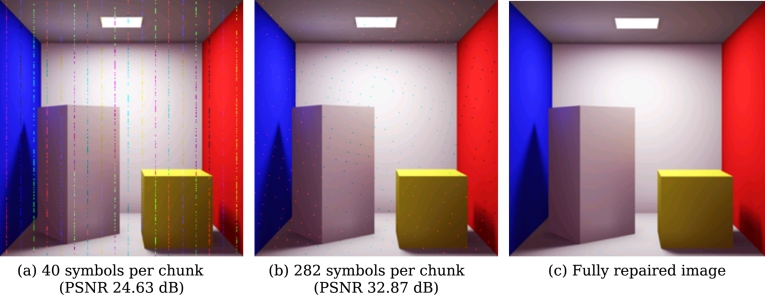
Cornell box image (240x240x16, CC0) encoded using NOREC4DNA. For (a), the width (multiplied by the depth per pixel) of the image was a multiple of the number of symbols in each chunk (40), leading to vertically aligned artifacts. In (b), with 282 symbols per chunk, the location of the propagated error was visually shifted. Since the encoding of (a) resulted in a considerably larger number of packets, the error propagated to more locations, resulting in a noisier image compared to (b). Figure (c) shows the output of the repair after 9 pixels of (b) were manually repaired/tagged.

Fig. 3 shows the impact of a 3-base substitution of a single line introduced to two different encodings. Fig. 3 shows a specific scenario where the width of the image is a multiple of the number of symbols per encoded chunk.

Using both the position and the *δ* required for reconstructing the errors, it might be possible to automatically correct any remaining errors in the decoded data. For this task, multiple solutions could be generated (if there still exists more than one possibly corrupted packet for an error), or the correct solution might be automatically recovered. In situations where an error is in a region where it is undetectable (e.g., if the error did not propagate to the header section), our implementation allows users to use a visual editor or upload a partially corrected version of the image. In the visual editor, the user marks pixels as incorrect, and the algorithm translates these pixels to positions in $b^{\prime }$ to tag the corresponding row (and column). With a sufficient number of tagged errors, the corrupted packet, including the erroneous position, can then be calculated. The user would then only have to correct any single corrupted region to repair the entire file. Using this approach, users can leverage the connection between the decoded rows to repair regions that might be harder to repair than others (e.g., the plain white area in the image of Fig. 3 vs. regions with multiple differing colors). Alternatively, using the upload feature, a user could repair various (simple to fix) regions of the image and upload the corrected version to automatically calculate the delta compared to $b^{\prime }$. For this purpose, image manipulation tools like GIMP or Photoshop can be used to automatically correct small regions of such a file using advanced techniques such as inpainting. The approaches of Pan et al. [34] and San Antonio et al. [38] demonstrated that techniques such as automatic anomaly detection and inpainting for images stored in DNA are generally feasible. Including such an approach in a future version of our approach could further increase the recovery chances for digital images stored in DNA. Similar to the detection of errors in plain text, this approach would counter the problem of false-positive guesses by using a consensus mechanism to find the most common row(s) in $b^{\prime }$, which were detected as having an anomaly.

#### 2.3.3 ZIP archives

While the previous file type-specific algorithms operate on files with a typically low information rate or a low (conditional) entropy and thus have a high likelihood of automatic or manual repair, we developed a method for ZIP archives. This method demonstrates that combining additional information introduced through the encoding can, to some extent, help to repair files with a high information rate. Due to their widespread use for compression for various file types, such as Office Open XML or JAR, ZIP archives are an ideal candidate to demonstrate the general usability of our approach. As a general-purpose compression method, this process could be applied to any input file format. However, employing more specific error detection algorithms tailored to the characteristics of the input file format is likely to yield better performance.

**Fig. 4:**
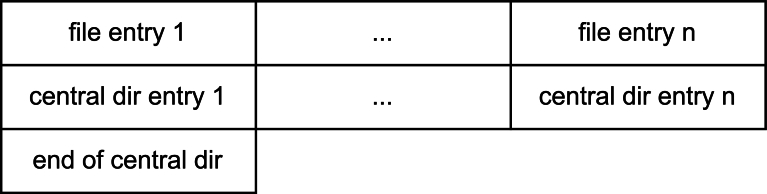
Structure of a ZIP file. Each file entry contains a file header as well as the compressed data.

As shown in Fig. 4, ZIP files contain several sections with various data points, some of which are either fixed, guessable, or redundantly stored. This allows the parser to check multiple fields in the file for correctness. Each compressed file is stored using a file header containing a header signature, the minimum version required for extraction, a general purpose flag, the compression method, the file modification date and time, the CRC-32 of the uncompressed file, the compressed and uncompressed size, the length of the filename and of the extra field, along with the filename and the extra field. Following this header, the actual data is stored. After all file entries, a central directory entry is stored for each file inside the archive. This central directory entry contains a header signature, the version this file has been compressed with, the minimum version required to extract, the general purpose bit, the compression method, the modification date and time, the CRC-32 of the uncompressed file, the compressed and uncompressed size, the file name length, the extra field length, the length of the file comment, the disk number where the file starts (0 in nearly all cases), internal and external file attributes, the relative offset of the local file header, the file name, the extra field as well as the file comment. After the central directory entries, a single end of central directory record exists, containing a signature, the number of disks, the disk where the central directory starts, (typically 0 or *0xFFFF* for ZIP64) the number of central directory records on this disk, the total number of central directories, the size and offset of the central directory, the length of the comment as well as the comment. Notably, there is a significant overlap between the fields of the file header and the corresponding central directory section. Using these fields allows us to tag specific areas inside the decoded data as valid or invalid. In the case of a mismatch between the two versions of a field, determining the correct version is not always straightforward. Two alternating candidates arise, one treating the field inside the file header as incorrect, and the other treating the field inside the central directory as incorrect.

Since the amount of additional information might not be sufficient to tag all bytes in a row in $b^{\prime }$ as correct, the calculation of the corrupted packet can be performed using only the columns in which an error was identified (or an error candidate, as described above). Assuming that columns i,…,k of the row *e* in $b^{\prime }$ were identified as corrupted, then the calculation of the corrupted packet would consider any row *l* as valid if $\forall_{x\in i,\ldots ,k}err[l,x]=0$ and invalid if $\exists_{x\in i,\ldots ,k}err[l,x]=1$, where *err* is the error matrix with the same dimension as *b* initialized with −1 (unknown state). Since the error introduced by a corrupted packet will always be in the same column(s), if there are multiple alternating candidates as described above, a viable approach to determine which of these alternatives is actually introduced by the corrupted packet would be to count the number of correct and incorrect rows for both combinations.

To parse the decoded ZIP file, we utilized a modified Kaitai Struct [36] parser. Since this parser may encounter failure due to a mismatch between expected and actual values, we implemented an on-error maximum likelihood repair. This approach allowed the parser to continue reading the file while simultaneously providing a corrected version of the file. If an error occurred in a length field, the parser would not detect the error and would most likely yield an error at the next mismatch. This could occur either during the attempt to decompress the payload or while expecting a signature at the wrong position. To address this issue, we introduced an additional parsing routine. This routine scans the file for all possible signatures (file header, central directory header, as well as the end of central directory header) and initiates the parser with the corresponding offset. This ensures that a mismatching length field will not truncate a succeeding header region, unless the header signature itself also contains an error. Furthermore, for each central directory entry, the offset to the local header is utilized to parse any undetected local file sections.

If multiple invalid packet candidates exist, the reduced number of possible packets and columns that might contain an error allows a brute-force repair. This is achieved by applying the known correction to all possible positions, while reparsing the new ZIP file to confirm correctness.

**Fig. 5:**
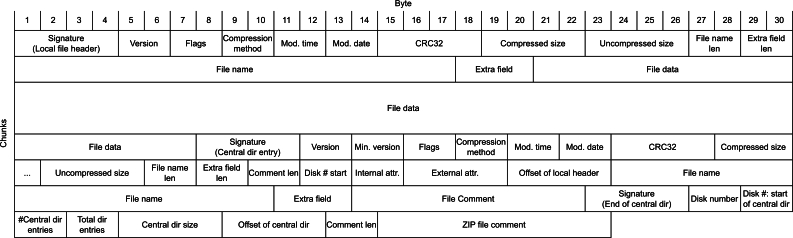
ZIP archive of a single file aligned to 30 byte chunks to represent the input / output of a fountain code. Depending on content and the number of chunks, redundant information stored in the local file header and the central directory header will be at different columns in *b*′

Fig. 5 shows the alignment of the various headers in the *b* matrix, dividing the file into multiple chunks. As long as the redundant information is not aligned in the same column in different chunks, an error in either position can be detected. If all matching fields align, an error can still be detected if only one of the chunks has been reduced using the corrupted packet. With an increasing number of files inside a ZIP file, the chance of gaining no information about the correctness of a chunk is significantly reduced.

#### 2.3.4 Manual or tool-based external (partial) reconstruction

In contrast to the previous three file type-specific methods, the reconstruction method presented in this section can be used for any file type that satisfies one of the following conditions: (a) a user can manually repair sections of the corrupted decoded file, or (b) an external (forensic) recovery tool can be used to (partially) repair the corrupted decoded file. To facilitate this, we provide an upload-based repair plugin that enables a more general approach to repairing corrupted files by detecting and reconstructing a subset of errors in the decoded file using third-party tools. Using the results of such tools, it is then possible to calculate the corrupted packet(s) and propagate the error delta to all affected rows in $b^{\prime }$, thereby addressing all remaining errors. This plugin allows users to manually (partially) repair the decoded data or to employ any (file type-specific) forensic tool to analyze, tag, and repair sections of the decoded file. Subsequently, the upload functionality can then be used to detect the changed content and exploit the chunk relationship introduced by the encoding to fully reconstruct the file.

#### 2.3.5 Manual missing data recovery

In this section, we present a method for missing data recovery in the case of a decoding that is not fully solved. This method is not restricted to the three file types described above and can thus be used for any encoded data.

If r=rank([A|b])<N for the received equation system Ax=b with $A\in F_{2}^{M\times N}$, then the equation system has free variables and thus (infinitely) many solutions. In contrast to a system with rank(A)=rank([A|b)=N, using Gaussian elimination to transform the augmented matrix [A|b] into reduced echelon form will not result in the left part of the matrix being the identity matrix. However, reducing only solvable lines during the Gaussian elimination results in a partially solved equation system $\left[A_{p}^{\prime }|b_{p}^{\prime }\right]$, where any row *i* is solved if row *i* in $A_{p}^{\prime }$ has exactly one 1 at position *i*. For this purpose, we perform an initial partial pivoting to ensure that (if possible) for each row *i* in *A* A[i,i]=1. After this step, any row *j* that does not meet this requirement will be inactivated. Additionally, the reduction of any column *j* will be skipped. This leads to a partially solved equation system, for which only rows with exactly one 1 (at position A[i,i]) will be treated as solved. This is the case if not enough packets were received to fully decode the stored information. All solvable rows will be directly returned, whereas any unsolved row will be treated as a null-row. In contrast to the previous scenarios, our method can only guide the user to which rows are missing. Using this approach, a user might be able to find an unsolved row that can be reconstructed manually. Furthermore, knowledge about the file structure and content might be used to infer the content of any missing row of the partially decoded file. Once such a row has been (partially) restored, the methods presented earlier can be used to correct any errors introduced by an incorrect reconstruction. With this approach, smaller incorrectly reconstructed parts can be treated as existing errors and thus can be corrected accordingly. In this case, the corrupted packet and all affected rows are already known, and only the error delta has to be calculated.

If no such manual reconstruction of a row is possible, only fully reduced rows can be retrieved and stored as a file in which not-decoded rows will be replaced with zero-bytes.

### 2.4 Implementation

**Fig. 6:**
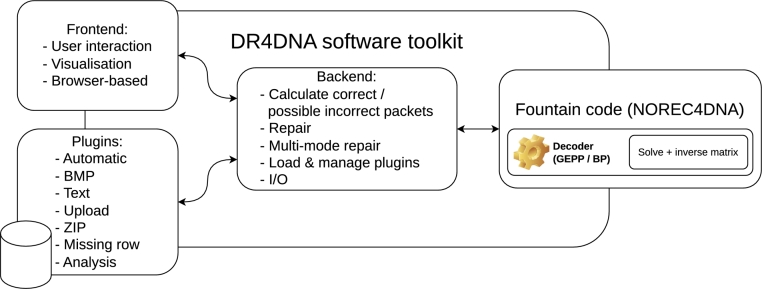
Overview: Components of the DR4DNA software toolkit. The backend manages the communication between all plugins, the frontend, and the used fountain code. Any compatible plugin present in the plugin folder will be integrated into the software toolkit.

We have implemented the methods described above in the DR4DNA software toolkit. The error detection and correction methods are implemented as plugins, which are automatically enabled if the requirements defined for a particular method are fulfilled. Furthermore, DR4DNA includes plugins for three file types while supporting the creation of additional generic or file type-specific implementations using a plugin system. These plugins will be automatically loaded if the analyzed file type is supported (via filename / -extension stored in the header-chunk or using static file analysis tools like ‘libmagic’ [16]). Alternatively, a user can also manually load such a plugin or use external software to partially recover the corrupted file and use the upload feature to propagate the changes as error deltas to the repair algorithm. An overview of the components of the proposed software toolkit is shown in Fig. 6.

Functions are integrated in DR4DNA that can be used whenever no single packet can be identified as an error source or if there are multiple possible error deltas for a packet. By implementing error correction as a plugin, a user can use all existing functions of DR4DNA, such as generating all possible solutions for a set of possible corrupted packets with known error deltas. Further information about the implementation and installation of DR4DNA can be found in the supplement.

## 3 Results

The encoded packets of a fountain code can be defined as an (over-determined) linear equation system (see Theorem 1). Our novel decoder-based method for error detection and correction exploits the structural characteristics of fountain codes. It effectively addresses errors occurring in packets that disrupt the consistency of the decoder's linear equation system. Solving such inconsistent equation systems using Gaussian elimination (or belief propagation) produces different results based on the order of the rows and the algorithm used (see Theorem 4). For a solved yet erroneous equation system derived from packets encoded using fountain codes, it is possible to calculate the index of a corrupted packet by tagging a sufficient number of rows as correct or incorrect (see Theorem 5). This tagging may either be performed manually by a user or automatically in the case of an over-determined and inconsistent equation system. Once the corrupted packet is known, a correction of the decoded file can be performed by correcting a single erroneous row affected by the corrupted packet during decoding. This process may either be performed by manually correcting such an erroneous row, thus retrieving the error delta, or by using two differing versions of the inconsistent equation system to automatically calculate the error delta in the case that the error produces an inconsistent equation system. Using the difference (i.e., the error delta) between the corrected and erroneous row, it is possible to directly repair a corrupted packet. If all chunks are reduced using the corrupted packet with the same error compared to their initially encoded version, the original file can be automatically reconstructed if a non-zero difference between multiple versions of the same (decoded) chunk can be calculated (see Theorem 7). Thus, our proposed decoder-based method for error detection and correction can detect an error as well as its delta to the original data to correct the error, even in the absence of additional dedicated error correction symbols.

Considering that the conditions required for this method are not always satisfied, our decoder-based approach can be complemented by our file type-specific and content-based error detection and correction methods. The latter helps users to identify corrupted packets and determine the error delta necessary for repair, minimizing the pool of potentially corrupted packets and error deltas, i.e., generating all remaining possible combinations for the encoded file can be a viable solution. When combined with a file-wide checksum, this automates the recovery of the originally encoded file. In contrast to the classification of entire chunks into valid and invalid, file type-specific and content-based error detection and correction methods operate at the byte level. The file type-specific reconstruction leverages cross-chunk dependencies introduced by the packets used for reduction during decoding. Thus, even a partially filled byte-level error matrix can substantially reduce the set of possibly corrupted packets or even enable a fully automatic recovery. We present such methods for three distinct file types: (a) plain text files, (b) BMP images, and (c) ZIP archives (see Section 2.3). Additionally, we discuss how to leverage external file recovery tools that may partially recover a file, on which our methods can then be applied to repair any remaining errors.

Thus, we distinguish between three operational recovery modes: (1) file-independent automatic recovery by using only the inconsistencies in the fountain encoding, (2) automatic recovery using file type-specific information and general knowledge about the stored data, and (3) guided manual recovery using both file type-specific information and the general knowledge about the stored data.

It is important to note that by using DR4DNA, no knowledge about the coding structure or the underlying algorithm is required. This isolates the previously described methods from their usage, allowing anyone to perform recovery operations. A researcher working on DNA storage systems utilizing any currently supported coding scheme based on fountain codes could thus directly use DR4DNA to perform recovery on otherwise corrupted data. The following experiments showcase usage scenarios, in which DR4DNA was used to repair corrupted data. While we highlight the underlying aspects involved in the process, recovery could be performed without prior knowledge about the code structure or the algorithms introduced in this article.

### 3.1 In-silico experiments

The results of several in-silico experiments that we performed to demonstrate the feasibility of our approach are presented below.

*Experiment 1*  In a first experiment, we demonstrate that our decoder-based method can be used to automatically recover encoded data in the presence of corrupted packets during decoding. For this purpose, we encoded the English text of the *Sleeping Beauty* novel [4], which we split into 331 chunks (including 1 header chunk), using 341 unique packets. To simulate a typical DNA storage scenario, each packet was appended with a 2 byte Reed-Solomon code, resulting in an encoded sequence length of 96 nt. During packet creation, only packets that adhered to the following rules were selected: (1) a maximum homopolymer length of 3, (2) a global GC content between 40% and 60%, and a GC content between 30% and 70% for each 50 nt window, and (3) no sequences containing a series of reserved motifs, such as primers and adapters, as well as their reverse complements. To simulate the DNA data storage channel, we used the MESA [39] framework. We selected the following settings for this simulation:

• Synthesis: ErrASE synthesis [21]
• Storage: Jukes-Cantor model [20] with q=0.001
• PCR: Pwo-Polymerase [30]
• Sequencing: Single End Illumina sequencing

Although some of these settings may not be widely used, they all represent reasonable upper bounds regarding error probabilities in their respective field. Additionally, these settings are also integrated, validated, and published in the MESA DNA storage simulator, enabling comparable and repeatable results [39]. This allows us to provide a rough worst-case estimate for more recent technologies.

Following random reordering, the resulting sequences were decoded. During decoding, 265 packets were identified as correct, 70 were successfully repaired by the Reed-Solomon code, and 6 corrupted packets were detected and consequently discarded. Notably, one of the seemingly correct packets that contained more errors than the Reed-Solomon code could reliably detect compromised the decoder's output. Since the errors in the packets left the linear equation system in an inconsistent state, our decoder-based method automatically recovered the correctly encoded data. Simultaneously, the (partial) reordering used in our decoder-based method (see Theorem 7) was able to recover the correct version of the file by creating all possible solutions and employing the CRC checksum included in the header to determine the correct version.

*Experiment 2*  In a second experiment, we demonstrate that combining our decoder-based method with our file type-specific and content-based method for plain text files can repair errors that our decoder-based method alone cannot repair. For this purpose, we used a random subset of 336 sequences from the first experiment, which contained a corrupt packet that did not produce an inconsistent equation system, rendering automatic recovery unattainable through our decoder-based method alone. Combining our decoder-based method with our file type-specific and content-based method for plain text files (see Section 2.3.1) to analyze the first 14 decoded rows, we were able to repair all errors introduced by the corrupted packet. Using only 14 rows to automatically tag correct and incorrect rows reduced the computation time and lowered the probability of false-positives, which seemed to occur for the chosen *Sleeping Beauty* novel due to the old English grammar and vocabulary. In the case of such false-positives, manual verification of the offending rows raised by our file type-specific and content-based method for plain text files may be required.

Lastly, the removal of an additional packet resulted in the inability to fully solve the equation system. Subsequently, a substantial portion of chunks remained undecoded, visually indicated in Listing 1 with the color yellow and dots representing an unsolved row:

**Listing 1:**
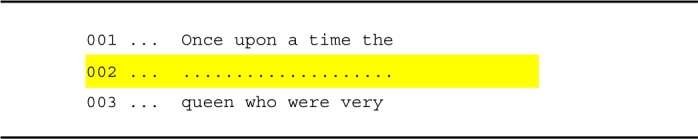
First three lines of partially decoded content. Manually adding a single missing row allows the decoder to complete.

By inserting a packet mapping directly to chunk 2 (therefore having a degree of 1) and the content “*re lived a king and* ” to the decoder, we were able to reconstruct all missing rows. Using our file type-specific and content-based method for plain text files, we could then automatically repair the remaining errors.

*Experiment 3*  In a third experiment, we demonstrate that our file type-specific and content-based method for ZIP archives cannot be used to automatically recover an original file, but our decoder-based method can successfully repair the error. For this purpose, we divided a ZIP archive containing 6 Python files, an image, and the text of the *Sleeping Beauty* novel into 609 chunks (including 1 header chunk) and encoded it into 914 packets. All encoding and simulation parameters were equal to those of the first experiment. After the error simulation, 657 sequences of correct length remained. This introduced a single corrupted packet to the decoder. The location of the error, occurring at the sequence's beginning, changed the seed, leading to a substantially different packet. Since this packet corrupted every byte in an affected row and impacted the majority of the ZIP headers, our file type-specific and content-based method for ZIP archives could not automatically recover the original file. However, since the rank of the linear equation system was smaller than the rank of the augmented system, our decoder-based method described in Section 2.2 could successfully repair the error.

*Experiment 4*  In a fourth experiment, we demonstrate that our file type-specific and content-based method for ZIP files can be used to guide the user towards manually identifying and tagging rows of a file as either correct or corrupted, such that all corrupted data in the file can be recovered. For this purpose, we split a ZIP archive containing 17 files, including the *Sleeping Beauty* novel, an image, and several Python programs, into 2,120 chunks to generate 3,180 encoded packets of length 180 nt. Each sequence included a three-symbol Reed-Solomon code. We simulated the storage process of the sequences using the MESA framework as described above, with the exception that the Jukes-Cantor model using q=0.1 was used to further increase the overall error probability and thus to simulate a more error-prone storage scenario. This process introduced a total of 2,928 errors. During decoding, 718 sequences were discarded due to non-repairable Reed-Solomon errors. One sequence contained a total of 4 mutations that were undetected by the Reed-Solomon code and thus included in the decoding process. Due to the fountain code structure, these errors were propagated to 1,105 rows. Our file type-specific and content-based method for ZIP files was able to guide the user towards identifying and tagging a total of 22 rows as either correct or corrupted. This was mainly performed by comparing the file names stored in each file header with those stored in the corresponding central directory header. This approach enabled us to correct all 4 errors by successively correcting two different rows containing corrupted file names.

*Experiment 5*  In a fifth experiment, we demonstrate that our file type-specific and content-based method for ZIP archives can be used to automatically reconstruct a ZIP file. We used a ZIP file containing only 2 Python files stored into 102 chunks. The overhead was chosen such that the resulting erroneous packets did not result in an over-determined linear equation system, where an automatic repair might have been possible. The information obtained through the file type-specific and content-based method for ZIP archives was sufficient to accurately derive the delta necessary to repair the error, while also reducing the number of possible corrupted packets to 18. This led to an automatic repair approach, where each resulting file was parsed and checked for correctness using the checksum included in the header chunk. The encoded ZIP archive was successfully retrieved automatically.

### 3.2 In-vitro experiments

To test our approach with in-vitro data, we used data from our previous in-vitro experiments conducted during the evaluation of DNA-Aeon [48]. This also demonstrates that our presented methods can seamlessly be combined with various fountain code based schemes.

Although the initial preprocessed entries in the FASTA file containing the DNA nucleotide sequences were randomly ordered, DNA-Aeon adds a metric indicating the correctness to its processed data. This metric allows the decoding process of the Raptor code to prioritize packets sorted by correctness, minimizing the likelihood of utilizing corrupted packets unless there is no suitable substitution. For the DNA-Aeon code, this implies that if an error occurs, it is less likely to increase the rank of the augmented matrix, rendering our decoder-based method less capable of error detection.

In our experiment, we selected the raw data of the 4.8 KB text file containing the German version of the *Sleeping Beauty* novel, which we split into 176 chunks (including a header) and encoded it using DNA-Aeon with a CRC interval of 5, synthesized, amplified, and sequenced. The raw data can be retrieved from the sequence read archive under the code SRR19954693 [48]. Given that the result of the original in-vitro decoding was error-free, we further reduced the coverage of the raw sequenced reads to approximately 0.01% of the raw total reads by utilizing random sampling. We performed the reduction in coverage using the identical sampling technique as described by Welzel et al. [48].

We processed the raw reads using a modified version of the Natrix pipeline [47], called RepairNatrix [41]. We used a mean quality threshold of 10 and a PANDAseq quality threshold of 0.3. The resulting FASTA file contained 313 sequences. We processed it using the DNA-Aeon decoder. Finally, we redirected the output of the inner code to be processed by our decoder-based method. With a rank of 175, the resulting equation system was under-determined and thus, as indicated in Listing 2, 97 rows could not be restored.

**Listing 2:**
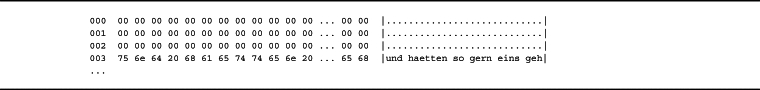
The state of the first three lines after a partial decoding. The first rows are not fully solved as indicated by the all zero row.

By manually inserting any missing row into the equation system, our decoder-based method was able to recover all missing rows. In this particular experiment, we opted to insert data for the first row, which contains the header information added by the fountain code. This header contains the length of the last chunk, the filename, optionally a file-wide CRC checksum, and a zero byte padding. Given the relatively low entropy of this row, we introduced a packet for this row that contained only zero bytes.

Listing 3 shows errors in the resulting text, but most of the reconstructed data is correct. Leveraging multiple iterations of our file type-specific and content-based method for plain text files (see Section 2.3.1), we could repair all remaining errors and retrieve the correct version, as shown in Listing 4. Alternatively, by definition, all initially missing and now corrupted rows can be treated as equal with respect to further corrections. Therefore, a user could have chosen any (or multiple) corrupted row(s) and applied appropriate corrections to reconstruct the original file.

**Listing 3:**
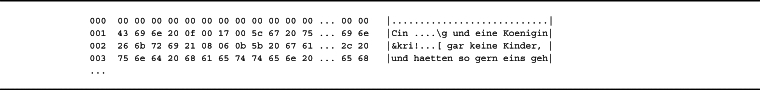
First three lines of the decoded data after an empty packet mapping to the first row was added to the equation system. A large portion of the previously missing data can now be viewed.

**Listing 4:**
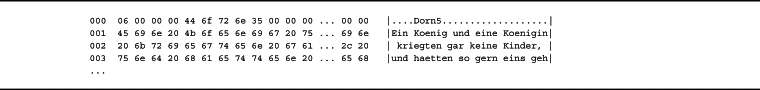
The first three lines of the fully recovered file after automatic or manual recovery repaired the remaining errors.

### 3.3 Applicability

**Fig. 7:**
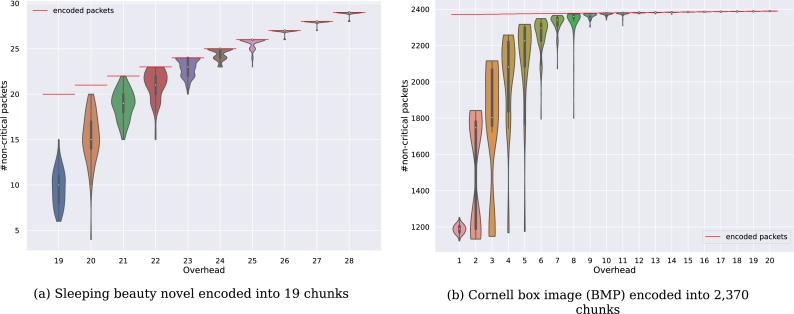
Number of non-critical packets for a given overhead. The files are encoded using the Raptor encoding of NOREC4DNA [40] with a sequence length of 300 bp. Non-critical packets, denoted by *p*_*n*_, are packets for which a subset *S* of packets in [*A*|*b*]∖*n* exists, such that ⊻_*s*∈*S*_*s* = *p*_*n*_. An error in a non-critical packet results in an inconsistent equation system. As mentioned earlier, an error in such a packet can be automatically detected and repaired. For each overhead, 100 repetitions were performed, resulting in a total of 1,000 (a) and 2,000 (b) simulations, respectively.

For DNA data storage, the number of corrupted packets as well as the number of errors inside these packets depend on the actual biological storage conditions. Therefore, these factors can only be mitigated to a certain extent, such as by increasing coverage during sequencing or avoiding error-prone sequences. Consequently, the success of repair and decoding relies heavily on the coding parameters. Since our decoder-based method can automatically repair a corrupted packet if the linear equation system is inconsistent, it is crucial to estimate the likelihood of this scenario. For this purpose, we encoded the German text of the *Sleeping Beauty* fairy tale [15] into an increasing number of packets using 19 chunks (18 + one header). We removed each packet iteratively from the equation system. If the decoder successfully decoded the encoded file, a different solution without this packet exists. According to Definition 1 and Theorem 4, Theorem 7, the removed packet is deemed non-critical. Given a linear equation system [A|b], a row or packet is critical if removing it decreases the rank of the augmented matrix rank([A|b]). Fig. 7a illustrates the number of such non-critical packets averaged over 100 repeats per overhead, each using a different initial seed, resulting in a distinct set of encoded packets. The results show that an increase in overhead substantially reduces the number of critical packets, increasing the potential for automatic repair. Fig. 7a shows that for a single packet of overhead, 50% of all packets are non-critical, implying that errors in these packets could be automatically repaired using our decoder-based method.

Since the number of chunks might influence the required overhead and the general decodability, we conducted the same experiment using the Cornell box image (Fig. 3c) with 2,370 chunks (2369 + one header). Fig. 7b shows that the relative median values are roughly equal, indicating a constant robustness. Moreover, our analysis shows that starting with an overhead of 16 packets (equivalent to 0.67% additional packets for the Cornell box image in Fig. 7b), all repeats yield 100% non-critical packets. This suggests that it is safe to assume that for data encoded in DNA using the Raptor encoding schemes implemented in NOREC4DNA [40], an overhead of 15+i,i≥1 or more packets ensures the successful repair of encoded data using the automatic repair algorithm. This is valid if up to *i* packets are corrupted, provided they meet the requirements for multiple corrupted packets defined in Section 2.2.

Not included in Fig. 7b are 10 degenerated encodings, which produced less than 100 non-critical packets. Such encodings typically occur if (multiple) of the generated packets are linearly dependent (e.g., encoding the same combination of chunks using different seeds). To maximize the resilience of the encoded data, a common approach would be to create a set of encoded packets that maximizes the number of non-critical packets. Therefore, focusing on the maximum value for each overhead in Fig. 7a and 7b shows that with an overhead between 3 and 7 packets, an encoding without critical packets is possible.

**Fig. 8:**
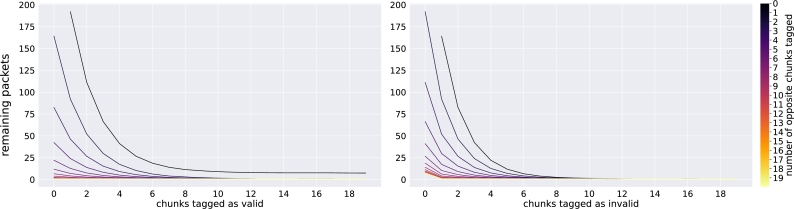
Number of possible packets remaining after chunks have been tagged as valid and invalid. In the first graph, the colored lines represent the number of chunks tagged as invalid. In the second graph, the colored lines represent the number of chunks tagged as valid. For this analysis, the *Sleeping Beauty* novel was divided into 166 chunks and encoded into 170 packets of length 168 nt. To mitigate outliers for both selected packets and chunks, the calculation was executed for each used packet where it was chosen as the corrupted packet. Then, for each combination of 0 to 20 chunks for the valid and invalid tags, 100 repeats were performed with random chunks chosen from the set of possible valid and invalid chunks for the selected packet. A total of 6,623,400 unique runs were performed.

Fig. 8 illustrates the impact of tagging rows in the solved equation system to identify a single corrupted packet. The graphs indicate that the impact of tagging chunks as valid to find a single corrupted packet has a slightly lower impact than tagging invalid packets. This observation is further confirmed by inspecting the experiments in which no invalid chunks were tagged. Here, the remaining packets could not be reduced to the singular invalid packet for any experiment performed.

**Fig. 9:**
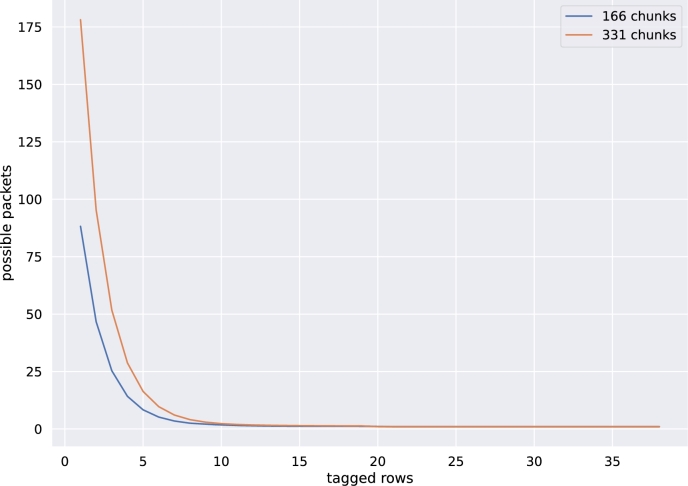
Comparison: average of the combined (valid and invalid) tagged rows required to reduce the amount of possible packets for the *Sleeping Beauty* novel encoded into 166 chunks vs. 331 chunks.

To verify that this behavior can be expected for larger values of the number of encoded packets, we repeated this experiment by encoding the *Sleeping Beauty* novel into 331 chunks, resulting in 338 encoded packets. As shown in Fig. 9, the graph for the experiment with 331 chunks is only marginally above the graph of the original experiment. In both cases, 16 tagged chunks are sufficient to reliably detect a single packet.

To successfully decode data encoded by any fountain code scheme, the following parameters are required: (a) the number of chunks the original file has been split into, (b) information whether a header chunk has been used, (c) the length of the seed, and (d) the length of the error correction code used per packet. However, given the limited amount of possible values each of these fields may have, an extensive search or a general convention (e.g., always using a header chunk) may be used to avoid having to transmit these values out of band. It is important to note that knowledge of these parameters is required independently of whether or not the proposed recovery methods are used.

## 4 Discussion

Our approach uses the underlying principle of fountain codes to (automatically) repair errors introduced during the DNA storage process without additional dedicated error correction schemes that lower the density of the medium. Pan et al. [34] improve file recovery in DNA storage systems by a machine learning approach for encoding and repair to recover corrupt image data after storing it in DNA, but this proposal is limited to image data (since image color channels are stored separately) and necessitates a custom coding method (to allow a machine learning algorithm to correct image color errors). Asteris and Dimakis [2] propose a repairable fountain coding scheme, but this scheme requires a modification of the code and does not take knowledge about the stored data into account. Furthermore, Asteris and Dimakis do not consider the particular properties of DNA storage. Jeong et al. [19] propose a DNA coding approach that relies on the LT-based encoding used by Erlich and Zielinski [10]. In this approach, (additional) error correcting codes as well as clustering are used to correct errors in encoded data. Our data recovery method can be used complementary to Jeong et al.'s approach. We provide data recovery in the case of a failed decoding and thus further improve the successful recovery of encoded data.

To the best of our knowledge, our work is the first to facilitate exact file recovery beyond the capability of the used code to detect and correct errors. It takes advantage of the fountain code structure to identify and use an inconsistent equation system and to perform (1) file-independent automatic recovery by using only the inconsistencies in the fountain encoding, (2) automatic recovery using file type-specific information and general knowledge about the stored data, and (3) guided manual recovery using both file type-specific information and the general knowledge about the stored data.

However, there is some potential for untapped optimizations. For example, the knowledge gained during the forensic analysis based on our method can be used to reduce the number of possibly corrupted packets and positions in these packets. Subsequently, an exhaustive search through all possible solutions could be performed. This would not be a fast or reliable solution, but in the realm of DNA storage, we assume that this could happen in the future where adequate compute resources are cheaper and more broadly available. This introduces a calculated trade-off between reduced synthesis costs by lowering the code-specific overhead and a potentially increased demand for computational power to recover a sequence in the event of an incomplete decoding. Our results show that the additional knowledge from the decoder can be used to reconstruct corrupted data. This, in turn, further increases the reliability of fountain codes for DNA as a storage medium while potentially lowering the cost.

### 4.1 Information density

One important aspect of DNA data storage is its information density, i.e., the amount of data that can be stored per nucleotide. With approximately 455 exabyte per gram [1], DNA as a storage medium can achieve a significantly higher information density than any current storage technology, while simultaneously offering a longer lifespan. While this metric only describes the physical density of the medium, the code rate as a metric for the percentage of actual information versus the total amount of data stored is a more suitable approach for defining the efficiency of a coding scheme. Traditional error correction codes such as Reed-Solomon [37] or LDPC [12] add redundancy that reduces the effective information rate. Some coding schemes for DNA storage use translations to convert the binary representation into a quaternary representation, while adhering to all channel restrictions, such as GC content and homopolymer count. This reduces the risk of insertions, deletions, and mutations, but it lowers the maximum information density and requires fine-tuning to achieve an adequate balance between error resilience and overhead.

In contrast, fountain codes are rateless erasure codes that do not operate with a fixed code rate. Due to their structure, they may require more or less overhead depending on the packets received. Modern fountain codes such as Raptor or RaptorQ [24], [32], [43] require only an average of (1+ϵ)n packets to decode a message that has been split into *n* chunks, where *ϵ* is the probability of failure. For Raptor and RaptorQ codes, an overhead of 1-2 packets is usually sufficient to achieve failure probabilities of less than 0.01% [24], [32]. To improve reliability, increasing the number of additional packets can further increase the resilience against erasure errors by being able to tolerate additional packet erasures. In the realm of DNA storage, fountain codes thus introduce only an overhead of *i* packets, *j* symbols per packet for the seed, as well as *k* symbols per packet for any optional error correction code. Given the two differing overheads of additional packets and the static per-packet overhead, the selection of a suitable packet length is a crucial parameter. For DNA data storage, the most efficient selection of the chunk size is determined by the synthesis method, which typically limits each packet to be stored in DNA to approximately 250-300 nt. Since the actual number of symbols per stored sequence depends both on the fountain code and the translation or inner code used, the information density varies. In the case of a plain mapping of the four bases A,C,G,T to the four possible binary numbers 00,01,10,11, no additional loss of information density occurs. However, for concatenated coding schemes such as DNA-Aeon [48], the byte-to-DNA base mapping may introduce additional overhead while also introducing redundancy, allowing for more efficient error correction.

It was shown that for the same amount of overhead, DNA-Aeon could outperform HEDGES [35] regarding constraint adherence and error correction capabilities. For encoding methods using fountain codes with a direct mapping, such as used in NOREC4DNA by Schwarz and Freisleben [40], high-scale random access on DNA storage systems by El-Shaikh et al. [9], or DNA fountain by Erlich and Zielinski [10], the information density can be directly estimated. Since fountain codes allow sampling packets until a sufficient set of rule abiding packets has been found, no additional overhead is required to ensure adherence to the DNA channel restrictions. Given the flat overhead of *s* bases per packet for a seed and an optional *e* bases for optional error correction codes per DNA strand, the information density *ID* per DNA strand of length *n* can be described as follows:

(7) $$ID=\frac{n-(s+e)}{n}$$

The overhead required for a successful decoding by modern fountain codes as Raptor or RaptorQ can be assumed to be static for larger numbers of chunks. For the DNA storage use case with its strictly limited chunk size and the rather large file sizes, this static overhead can be mostly ignored. Assuming a file size of 100 kB, a 4 byte seed, no error correction code, a DNA strand length of 300 nt, and a fixed overhead of 16 packets, the information density is 0.9360374269005848, which is close to the per-packet information density of $\frac{284}{300}=0.94\bar{6}$. For a file size of 1 MB, the information density rises to 0.9455925111694206 and for 10 MB to 0.946559138258248. When reducing the overhead of packets to 2, this value further increases to 0.9466532242796016. Since the seed is comparable to a unique identification required for the unordered DNA storage system, the usage of the seed does not introduce a considerably higher overhead compared to existing coding solutions for DNA storage.

Our proposed recovery method uses the coding structure in conjunction with the overhead required for fountain codes to perform error correction for this code family, which otherwise is limited to the resilience against erasures. No additional overhead and thus no reduction of the information density compared to previously published fountain code solutions for DNA storage [10], [40], [48] is required. However, as mentioned in Section 3.3, an additional overhead increases the probability of automatic repair, and thus the proposed recovery method is more efficient (a) when working with a large number of packets and (b) when additional packets are present.

The information density per DNA strand is the major factor in determining the overall information rate. Thus, the maximum reliable and cheaply available packet length should be used. As stated previously, currently commercially available synthesis solutions are most cost-efficient at approximately 300 nt per DNA strand. Since the bidirectional mapping from the DNA alphabet to binary is fixed per code and the length of each chunk in a fountain code is the same for all chunks and thus packets, the number of symbols per chunk can be directly inferred from the DNA strand length minus the length of the seed and any additional error correction.

### 4.2 Usage scenarios

To make our methods accessible to a broad range of scientists in the area of DNA data storage, DR4DNA is designed to be used by researchers with different backgrounds. For example, computer scientists or bioinformatics researchers can use, integrate, and extend the open source software provided by us to tailor it to their use cases and further improve the reliability of coding schemes based on fountain codes. Furthermore, DR4DNA's no-code approach and low parameter setup allows biologists to directly use our advanced recovery methods. Together with the provided simplified workflow, intuitive visualizations, and the user-friendly plugin system, DR4DNA eases the DNA storage process, allowing biologists without in-depth knowledge of fountain codes to not only use state-of-the-art coding schemes, but also recover the stored content in the case of an erroneous decoding. By decoupling technical coding details from automated or content-based recovery, it is possible to intuitively use the provided recovery methods without any expertise in coding schemes or advanced file recovery techniques. This allows a biological researcher to either automatically recover corrupt files or use knowledge about the stored content to repair errors in a file, without requiring a background in computer science.

### 4.3 Feedback-based decoding schemes

We have demonstrated that the presented methods can be used in combination with DNA-Aeon to correct erroneous data. The next step would be to create a feedback loop that allows DNA-Aeon to use the information obtained by our methods to increase DNA-Aeon's error resilience. DNA-Aeon employs NOREC4DNA to generate binary sequences of equal length, which are then converted to specially crafted codewords [27] using an arithmetic code. This code strategically inserts periodic checksums. The inner code's proposed decoder yields the packet with the highest likelihood to pass all checksums and to satisfy the channel restrictions defined during encoding (e.g., maximum homopolymers, GC content). Given that DNA-Aeon uses periodic checksums and the presented methods can, under certain restrictions, locate the error region, a feedback loop could empower DNA-Aeon to iterate through multiple versions of a checkpoint region to find a sequence that fulfills (1) checksum requirements, (2) channel restrictions, and (3) error detection criteria introduced by our methods.

### 4.4 File recovery by zero-byte padding

A different approach to harness the potential of the presented methods is to add artificial empty chunks to the input file during encoding. This lowers the information density, but these artificial chunks can be used to determine the source of an error as well as the error-delta required to repair the corrupted packet. For this purpose, each encoded packet would include precisely one of these additional chunks (randomly selected with an equal distribution) during its creation. Although this approach cannot be used to directly repair a corrupted packet (since it might not be possible to identify the offending packet), the delta required for a successful repair can be detected directly. The incorporation of multiple empty rows can further increase the sensitivity to an invalid difference when several corrupted packets impact a single added zero-row. If any packet contains an error, solving the equation system would yield an inconsistent equation system, as described in Theorem 4. This inconsistency arises either due to multiple possible solutions introduced by the corrupted packet or by adding the known zero rows to the equation system. The correct file can then be retrieved using the inconsistency of the equation system, as defined in Theorem 7. For multiple corrupted packets, this approach could detect an error as long as there exists a reduction for each added empty row *i* where *i* results in a non-empty row if any packet derived from this row is corrupted. Such a condition is met only when two or more corrupted packets impact a single added zero-row in such a way that the overall error-delta for this row would be a zero-vector. Furthermore, if the number of received packets *r* is smaller than the number of encoded chunks (including the number of added empty rows), the incorporated empty rows in the equation system could be used to trade the added error resilience for erasure resilience. This effectively eliminates the additional overhead introduced by this method.

Although the implementation of such coding techniques is beyond the scope of our current work, this demonstrates the potential integration of our error detection and correction methods into future codes for DNA data storage.

## 5 Conclusion

In this paper, we proposed a novel approach to provide data recovery for DNA storage. In particular, we presented a novel decoder-based method for automatically recovering corrupted data stored in DNA using fountain codes. This method acts as an error detection and recovery scheme, leveraging the unique structure of the fountain-encoded data for error detection and correction. Since the decoder-based method requires a corrupted packet to be non-critical and therefore only works for an inconsistent equation system, we developed additional file type-specific and content-based error detection and correction methods. Our current implementation covers error detection and repair methods for three file types. We demonstrated that the knowledge embedded in the encoding can be combined with file type-specific redundancy and general knowledge about the stored data to successfully reconstruct data from a partially corrupted DNA storage system. Since this file type-specific and content-based approach relies on the encoded file structure and its content, our approach helps to bridge the gap between DNA storage and conventional file systems, for which such file type-specific and content-based recovery methods exist. Therefore, our file type-specific and content-based recovery is inherently limited by the ability to analyze and predict the structure of the file and/or its content. Thus, encrypted files and files of unknown type or structure may not profit from the proposed file-type specific and content-based approach. Since our proposed automatic decoder-based method is applicable to any content under the preconditions defined in this work, and since any file can be compressed using ZIP, the ZIP-based plugin we introduced is a universal approach towards more resilient fountain code-based DNA storage systems. Furthermore, we evaluated the proposed methods using both in-silico and in-vitro experiments and discussed their limitations. The limitation regarding fully automatic recovery can be used as a recommendation for future encoding schemes using fountain codes to store information using DNA as a storage medium. Finally, we introduced DR4DNA, a software toolkit that incorporates all the presented methods, demonstrating the practical feasibility of our approach for researchers with or without prior knowledge in coding for DNA storage.

Future work could focus on refining the proposed file type-specific and content-based error detection and correction methods by employing advanced anomaly detection schemes. Moreover, a key aspect to consider in the future is the implementation of fountain code-based encoding schemes that directly incorporate the presented methods. This integration could enhance reliability, error resilience, and efficiency by, for instance, combining Reed-Solomon codes with information about corrupted packets and the most likely error positions. Using this information, the Reed-Solomon code could treat detected errors as erasures, effectively doubling the error-correcting capabilities. Further research could also explore the integration of other error correction techniques. An example is enzymatic repair, as proposed by Meiser et al. [31]. This approach is based on repairing chemical decay in the form of nicked DNA strands. By analyzing the enzymatic repair approach, researchers could obtain valuable information about the expected errors and their most likely locations. DR4DNA could then use this information to calculate any remaining errors, either automatically or through a feedback loop involving the user. Apart from improving the functionality of the presented recovery methods, future work should also include a user study to detect any shortcomings or unclear usage patterns, in particular involving biologists without in-depth knowledge of DNA coding schemes based on fountain codes.

## Inclusion and diversity

We support inclusive, diverse, and equitable conduct of research.

## CRediT authorship contribution statement

**Peter Michael Schwarz:** Conceptualization, Data curation, Formal analysis, Methodology, Resources, Software, Validation, Visualization, Writing – original draft, Writing – review & editing. **Bernd Freisleben:** Conceptualization, Funding acquisition, Project administration, Supervision, Writing – review & editing.

## Declaration of Competing Interest

The authors declare no competing interests.

## Data Availability

The source code for the created software is available at https://github.com/umr-ds/DR4DNA.

## Appendix A Supplementary material

MMC Additional information regarding usage and automatic correction of multiple errors. MMC
