# Supplementary material for: Data recovery methods for DNA storage based on fountain codes
Source: Comput Struct Biotechnol J. 2024 Apr 24;23:1808–23. doi: 10.1016/j.csbj.2024.04.048 (PMC11066528; doi:10.1016/j.csbj.2024.04.048)
Supplement: MMC — Additional information regarding usage and automatic correction of multiple errors. [file mmc1.pdf]

Installation and configuration of DR4DNA

Installation

The required code can be downloaded from https://github.com/umr-ds/DR4DNA as described in the accompanying README file.

Configuration

DR4DNA requires a *.ini* file created from NOREC4DNA, which is included as a submodule. Using this file, we recommend the following command to start DR4DNA:

```
$ python app.py <ini_file.ini>
```

DR4DNA usage

An manual for DR4DNA can be found in the GitHub repository.

Automatic correction of multiple errors

As shown in Figure 1, if there are multiple corrupted packets, it is possible to detect and automatically correct all corrupted packets (under the restrictions defined earlier).

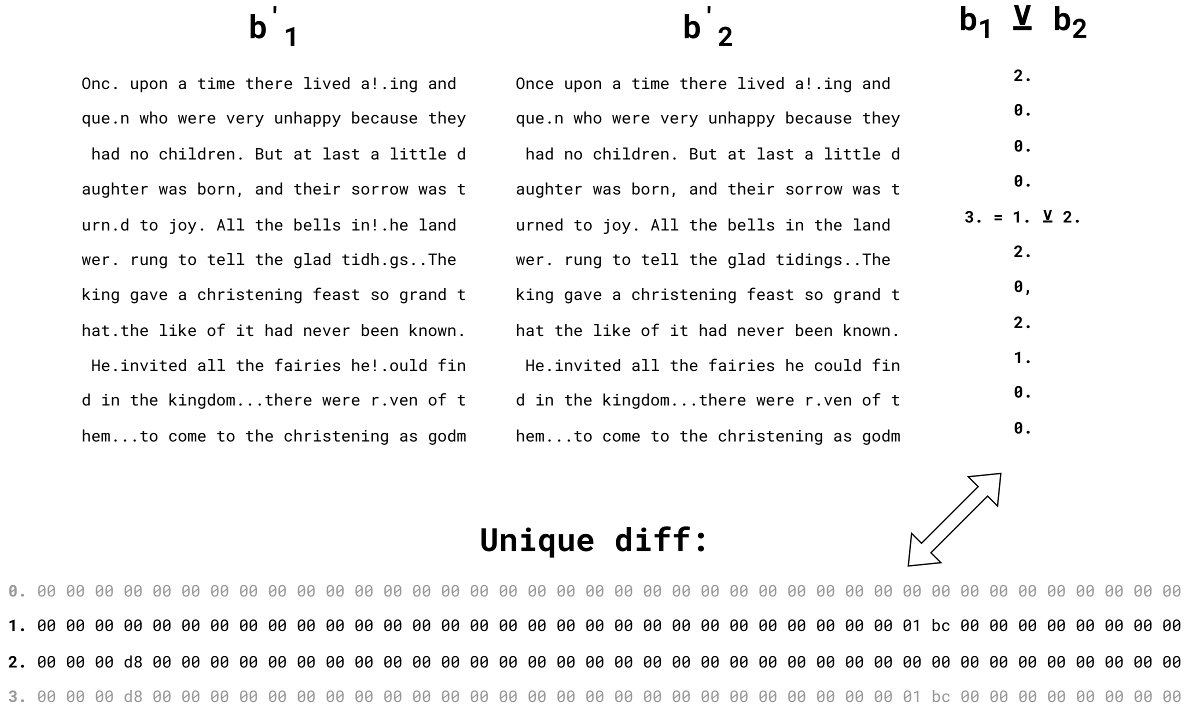

Figure 1: Overview: Automatic detection and correction of multiple corrupted packets. By comparing multiple permutations of the equation system, it is possible to identify all error deltas. The search for the corrupted packet has to be performed for each unique and irreducible error delta. If a comparison yields a reducible row (3.), the row can be used for all unique components (1. and 2.)

By analyzing the difference between the created solutions, it is possible to infer the potentially corrupted packets. A_1^{-1} as well as A_2^{-1} can be used to infer which packets were involved during the reduction of the current row. Inverting the permutation applied to the packets before decoding yields packets that can then be compared using set operations such as intersection, difference, and XOR.
